# Drug Development in Non-Oncogene-Addicted Non-Small Cell Lung Cancer

**DOI:** 10.3390/cancers18050880

**Published:** 2026-03-05

**Authors:** Pedro Cruz, Cristina Boixareu, Diogo J. Silva, Joshua Ting, Rayssa Sena, Steph A. Pang, Stephanie Mullings, Anna Minchom

**Affiliations:** Drug Development Unit, The Royal Marsden Hospital and The Institute of Cancer Research, London SM2 5GP, UK

**Keywords:** carcinoma, non-small-cell lung, clinical trials, drug development, antibody–drug conjugates, immunotherapy, non-oncogene-addicted non-small cell lung cancer

## Abstract

Non-oncogene-addicted non-small cell lung cancer therapy has seen major advances in recent years. New molecular targets and biomarkers have enabled the development of drugs as diverse as immunotherapies and antibody–drug conjugates, among others. With a pharmacological armamentarium so precise, phase I trials have also evolved from exclusively toxicological studies into early efficacy signal-seeking trials. Nonetheless, difficulties remain, with the frequent failure of new drugs when progressing to a phase III setting. Challenges are seen in the setting of later lines therapy (testing against docetaxel), of which there are several examples. These are being tackled with promising new drugs being developed, based on innovative biological rationales. We review the current state of the art of drug development in non-oncogene-addicted non-small cell lung cancer, including advances, new drugs and targets, challenges, and opportunities in drug development.

## Simple Summary

Non-oncogene-addicted non-small cell lung cancer therapy has seen major advances in recent years, with more precise and diverse drugs. Phase I clinical trials have also evolved. Nonetheless, difficulties remain, with the frequent failure of new drugs when progressing to phase III. These challenges are being tackled with promising new drugs being developed, based on innovative mechanisms. We review the current state of the art of drug development in non-oncogene-addicted non-small cell lung cancer, including advances, new drugs and targets, challenges, and opportunities in drug development.

## 1. Introduction

Lung cancer is a lethal cancer [1] with a 5-year survival rate of 27% [2,3]. Non-small cell lung cancer (NSCLC) represents 84% of lung cancers [4]. In 2022, there were 2,212,710 new NSCLC diagnoses worldwide [5]. The 5-year survival is 6% for those with stage IV NSCLC [4]. A significant minority of NSCLC cases have actionable genomic alterations (oncogene-addicted), including *EGFR* (epidermal growth factor receptor), *ALK* (anaplastic lymphoma kinase), *MET*, and others. Oncogene-addicted NSCLC outcomes have been improved significantly by the successive targeted therapy breakthroughs since the approval of gefitinib in 2003 [6]. Some oncogene-addicted patient groups have overall survival (OS) exceeding 47 months [7,8,9,10,11,12]. However, the same improvements in outcomes have not yet been afforded to the 40% of NSCLC that are non-oncogene-addicted, defined as those not dependent on an oncogene [13]. These have a heterogeneous biology, which can include genomic alterations currently lacking targeted therapy (e.g., *TP53*, *STK11* [serine/threonine kinase 11], and *KEAP1* [Kelch-like ECH-associated protein 1] mutations) [14], smoking-induced immunogenic neoantigens [15], and heterogeneous tumour microenvironment phenotypes [15,16,17,18,19,20].

This remains an unmet need. Pivotal first-line trials reported a median OS of 17.1 [21], 21.4 [22], 22.0 [23], 26.3 [24], 30.0 [25], and 34.5 months [26] according to subgroups, unsurpassed in other studies since [23,25,27,28,29,30,31,32,33,34,35,36].

With the increasing understanding of non-oncogene-addicted NSCLC biology (hereon referred to as NSCLC) and evolving drug technologies, new rationally designed therapeutic technologies are reaching standard of care, including immunotherapies, bispecific antibodies, and antibody–drug conjugates. As the pharmacological armamentarium is becoming more precise and diverse, phase I trials have started to seek early efficacy signals alongside toxicity profiles, and can even be argued to have therapeutic benefit for some patients [37]. Despite progress, challenges remain. Surpassing acquired resistance to the current therapies requires an increasing understanding of tumour biology and innovative drug design. Additionally, when moving from early to late phase development, new NSCLC drugs face attrition, as they often fail to show benefit in phase III trials, usually versus docetaxel in the second-line setting [38].

This paper aims to review the current state of the art of drug development in non-oncogene-addicted NSCLC. Considering the current standard of care options, we discuss recent advances in drug development, why advances may not lead to changes in the standard of care, and possible ways to overcome these challenges. A literature search was carried out, including articles in the PubMed/MEDLINE and Scopus databases, published up to January 2026. The search included various combinations of terms, such as “drug development”, “non-small-cell lung cancer”, “early phase”, “phase I clinical trial”, and “phase II clinical trial”. Articles were included according to their clinical relevance and level of evidence, emphasising randomised controlled trials, international guidelines, and expert reviews. Articles on early-stage and locally advanced NSCLC, oncogene-addicted metastatic non-small cell lung cancer, or written in any language other than English, French, Spanish, or Portuguese, were excluded.

## 2. Non-Small Cell Lung Cancer

**Figure 1:**
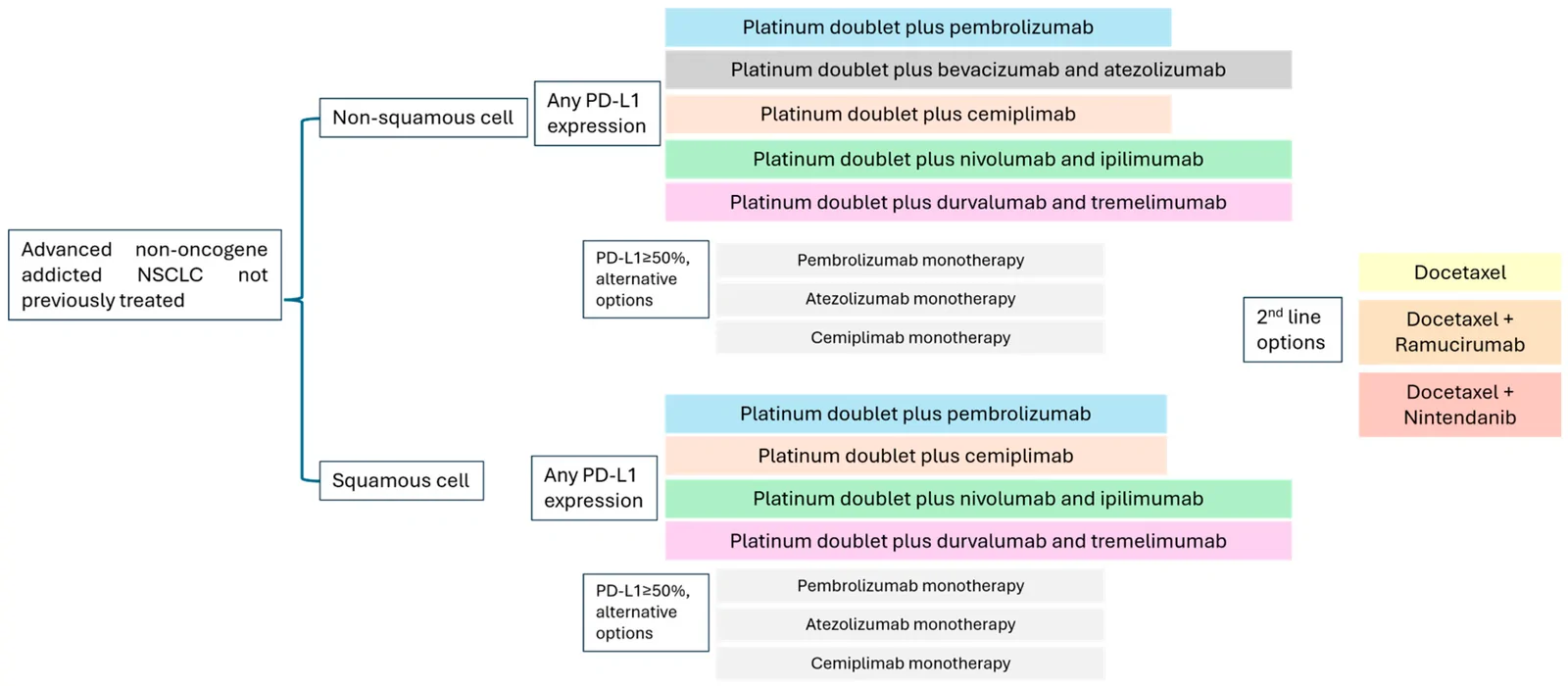
Current landscape of non-oncogene-addicted non-small cell lung cancer therapies. Abbreviations: NSCLC: non-small cell lung cancer, PD-L1: programmed death-ligand 1.

The standard treatment of non-oncogene-addicted NSCLC has evolved significantly in the last decade, particularly with the incorporation of immune checkpoint inhibitors, primarily targeting PD-1/PD-L1 (programmed death protein 1/programmed death-ligand 1) (Figure 1).

Before the immunotherapy era, the treatment of advanced lung cancer was largely based on platinum combination regimens. The ECOG 1594 trial [39] established platinum doublets as equivalent standards, including cisplatin plus paclitaxel, cisplatin plus docetaxel, cisplatin plus gemcitabine, and carboplatin plus paclitaxel. Subsequently, the combination of platinum plus pemetrexed was shown to be superior in patients with non-squamous histology [40].

The KEYNOTE-189 trial [41] (non-squamous NSCLC) established pembrolizumab plus platinum-based chemotherapy as the standard of care. It showed a significant survival advantage over chemotherapy alone (median OS 22.0 versus 10.7 months; hazard ratio [HR] 0.56; 95% confidence interval [CI]: 0.45 to 0.70), with benefits extending across all PD-L1 subgroups. KEYNOTE-407 [42] confirmed this benefit in squamous histology, with improved OS, progression-free survival (PFS), and overall response rates (ORR). Additional immune checkpoint inhibitor-based combination trials, such as EMPOWER-Lung 3 [43] (cemiplimab in combination with chemotherapy) and IMpower150 [44] (atezolizumab in combination with bevacizumab and chemotherapy), have further reinforced the efficacy of chemo-immunotherapy regimens, the latter being restricted to non-squamous histology. In the first-line setting, CTLA-4 (cytotoxic T-lymphocyte associated protein 4) targeting regimens like CheckMate 9LA [45] (nivolumab in combination with ipilimumab and limited chemotherapy) and POSEIDON [46] (durvalumab in combination with tremelimumab and chemotherapy) also showed survival advantages over chemotherapy alone. Immune monotherapy remains an option for selected patients with high PD-L1 expression (≥50%), based on KEYNOTE-024 [25], IMpower110 [47], and EMPOWER-Lung 1 [48].

Following progression on immunotherapy, treatment options remain limited. Standard second-line therapies include docetaxel, either alone or in combination with antiangiogenic agents such as ramucirumab [49] (REVEL trial) or nintedanib [50] (LUME-Lung 1). However, the benefits in PFS and OS are modest, with median OS slightly exceeding 10 months and PFS around 4 months. Nintedanib is an oral triple angiokinase inhibitor that targets vascular endothelial growth factor receptors (VEGFR) 1–3, fibroblast growth factor receptors (FGFR) 1–3, and platelet-derived growth factor receptors (PDGFR) α/β. In LUME-Lung 1, adding nintedanib to second-line docetaxel significantly improved median PFS 3.4 versus 2.7 months (HR 0.79; 95% CI: 0.68 to 0.92), median OS to 12.6 versus 10.3 months (HR 0.83, 95% CI: 0.70 to 0.99), and disease control rate (DCR) to 60.2% versus 44.0% (OR 1.93, 95% CI: 1.42 to 2.64) in patients with lung adenocarcinoma following chemotherapy [50]. These results were confirmed in VARGADO, a 10-year prospective, non-interventional, real-world study that analysed nintedanib and docetaxel in lung adenocarcinoma patients progressing after either immunochemotherapy or sequential chemotherapy and immunotherapy [51,52]. Ramucirumab is a human IgG1 monoclonal antibody that targets the extracellular domain of VEGFR-2. REVEL is a randomised phase III trial comparing ramucirumab plus docetaxel versus placebo plus docetaxel for second-line treatment of stage IV NSCLC after progression on platinum-based therapy. Median overall survival was improved to 10.5 months versus 9.1 months; HR 0.86; 95% CI: 0.75 to 0.98 [49].

## 3. Drug Advances in Non-Small Cell Lung Cancer

There are a plethora of targets and drug classes under investigation for non-oncogene-addicted NSCLC. Recent key advances are highlighted, especially regarding antibody–drug conjugates and immunotherapies.

### 3.1. Antibody–Drug Conjugates

**Figure 2:**
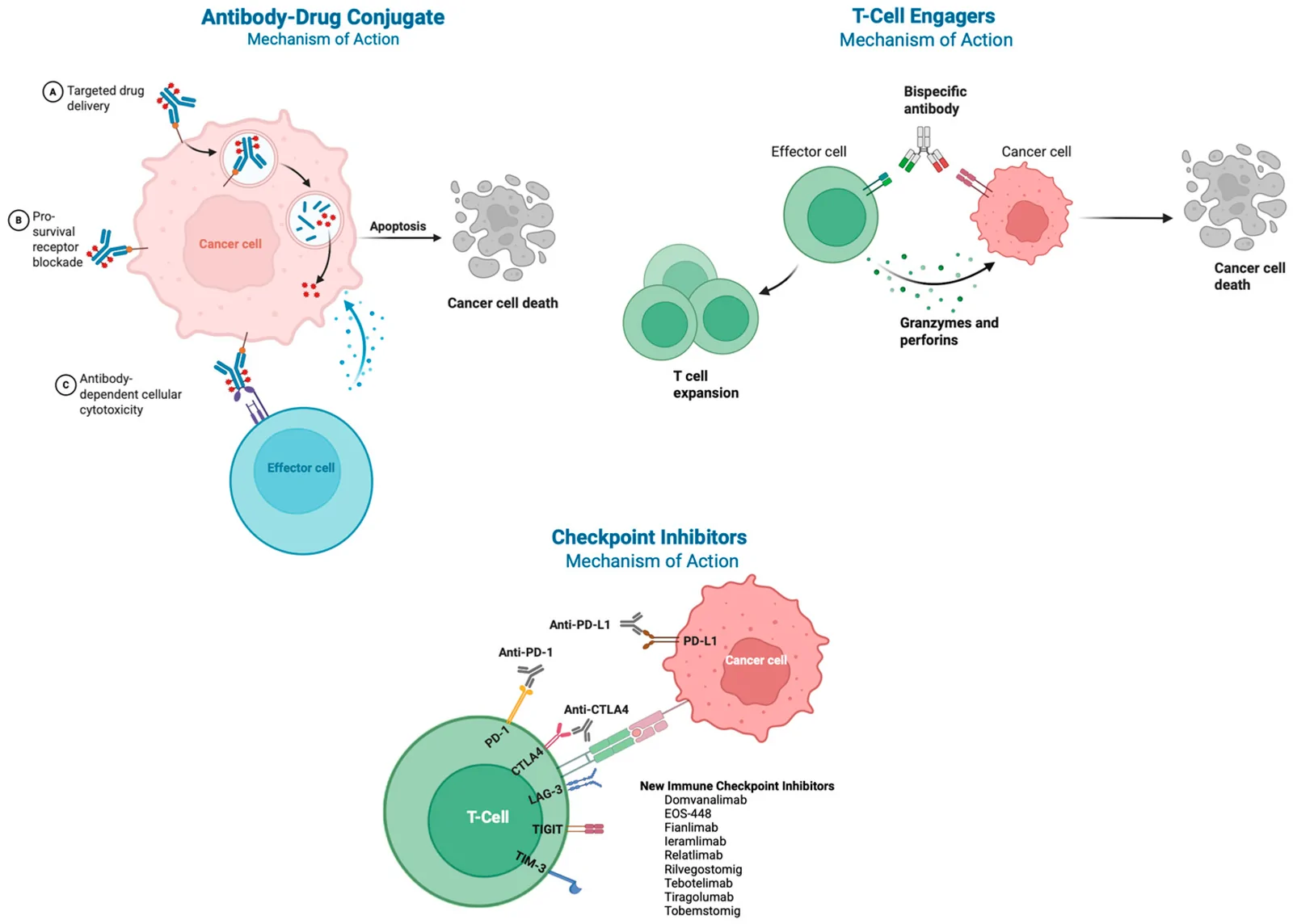
Drug classes in non-oncogene-addicted non-small cell lung cancer. Abbreviations: CTLA-4: cytotoxic T-lymphocyte-associated protein 4, LAG-3: lymphocyte-activating gene-3, MHC: major histocompatibility complex, PD-1: programmed death-ligand 1, TCR: T-cell receptor, TIM-3: T-cell immunoglobulin and mucin domain-containing protein 3, TIGIT: T-cell immunoglobulin and mucin domain-containing protein 3. Created in BioRender. Silva, D. (2026) https://BioRender.com/jakmmeo (accessed on 3 March 2026).

Antibody–drug conjugates (ADCs) are molecules that allow the selective delivery of a payload (usually a very high-potency cytotoxic), which is attached to an antibody via a linker [53,54]. Inactive in circulation, the antibody specifically binds its target on cancer cells and is endocytosed into a lysosome, where the cytotoxic payload is released [53,55], thereby minimising off-target side effects and increasing the therapeutic index [54,56] (Figure 2). The antibody portion of an ADC is responsible for directing the conjugate to its intended cellular target. Its binding affinity and specificity are essential, as they determine how effectively the ADC can recognise and attach to tumour cells while minimising interactions with normal, healthy tissues [57].

The linker must achieve an optimal balance between stability in circulation and efficient release at the target site. Linkers can be cleavable or non-cleavable. Cleavable linkers are engineered to respond to features characteristic of the tumour microenvironment (TME), such as acidic pH or the activity of lysosomal enzymes, allowing the payload to be selectively liberated once inside tumour cells. This design enhances targeted drug delivery and helps limit systemic exposure. In contrast, non-cleavable linkers remain intact until the entire ADC is internalised and degraded within the cell, at which point the payload is released through normal intracellular breakdown processes. If a linker is excessively stable, the payload may not be efficiently freed after uptake, diminishing therapeutic impact. Conversely, if the linker is insufficiently stable, premature release in the bloodstream can occur, increasing the risk of off-target toxicity. Some linkers may also carry a risk of immunogenicity or possess intrinsic toxicity. The most effective linkers are those that demonstrate high selectivity for tumour tissues, thereby reducing unintended damage to healthy cells and improving the overall therapeutic index of the ADC [58].

Effective target-driven internalisation is a key requirement for ADCs, as it enables the cytotoxic payload to reach the interior of cancer cells. Both the speed of uptake and the behaviour of the antigen–ADC complex as it moves through endosomal and lysosomal compartments directly shape how and when the payload is released. It is therefore important to understand whether a given antigen is mainly routed back to the cell surface or preferentially trafficked toward lysosomal degradation. Targets that recycle rapidly to the plasma membrane can limit ADC accumulation in lysosomes, ultimately reducing payload liberation into the cytosol and diminishing overall potency. Because of this, dynamic measurements of internalisation have become valuable indicators of ADC performance [58]. Additionally, antigen shedding, often mediated by tumour cell-produced proteases, can significantly impact the effectiveness of ADCs [57] and increase the risk of off-target toxicity due to non-specific binding [59]. The TME can influence the development of resistance to ADCs. Cancer-associated fibroblasts (CAFs) contribute to therapeutic resistance by altering and stiffening the extracellular matrix, which can hinder drug penetration [60]. Myeloid-derived suppressor cells (MDSCs) and tumour-associated macrophages (TAMs) within the TME release cytokines and growth factors that enhance tumour cell survival and proliferation, ultimately diminishing the effectiveness of chemotherapeutic agents [61]. Neutrophil extracellular traps (NETs), DNA-protein structures released by neutrophils in response to various stimuli, including tumour-derived signals, can form a physical barrier that obstructs the diffusion of agents [62], and can further establish a pro-tumour milieu by facilitating immune evasion and supporting tumour growth and survival through activation of pathways such as MAPK and NF-κB38, plus promote the recruitment and activation of additional immune cell populations [63]. In addition, hypoxic regions in the TME intensify resistance mechanisms by stabilising hypoxia-inducible factors that drive more aggressive tumour behaviour [64]. Beyond these mechanisms, the TME can directly induce cellular quiescence, rendering tumour cells less responsive to both cytotoxic and targeted treatments [65]. A deeper understanding of these interconnected processes will be crucial for the rational design of next-generation ADC payloads.

Many targets are under investigation for ADCs in NSCLC, the most promising detailed here.

HER2 (human epidermal growth factor receptor 2) is a membrane protein activated by dimerisation [66,67]. DESTINY-Lung02 was a multicenter, blinded, phase II study that enrolled 152 previously treated patients with HER2-mutant NSCLC. Patients were randomly assigned in a 2:1 ratio to trastuzumab deruxtecan 5.4 or 6.4 mg/kg once every 3 weeks. The 5.4 mg/kg dose achieved an ORR of 49%, a median DOR (duration of response) of 16.8 months, and a median PFS of 9.9 months, whereas the 6.4 mg/kg dose yielded an ORR of 56% and a median PFS of 15.4 months [68]. Adverse events of note included drug-related interstitial lung disease occurring in 12.9% of participants in the 5.4 mg/kg arm and 28.0% in the 6.4 mg/kg arm [68]. These results supported FDA and EMA approval of trastuzumab deruxtecan for activating HER2-mutant unresectable or metastatic NSCLC following prior systemic therapy [69]. This approval represents a practice change in the oncogene-addicted population, but given the cell surface expression of HER2 described in 12 to 78% of NSCLC and averaging 35% in a meta-analysis [70,71,72,73], including cases without HER2 mutations, trastuzumab deruxtecan has also been investigated in non-oncogene-addicted NSCLC. The DESTINY-PanTumor02 trial, an open-label phase II study, enrolled more than 260 previously treated patients with HER2-expressing (IHC [immunohistochemistry] 3+ or 2+ by local or central testing) advanced solid tumours. Among patients with centrally confirmed HER2 IHC 3+ expression (*n* = 75), the ORR was 61.3%, with a median DOR of 22.1 months and a median PFS of 11.9 months [74], contributing to FDA (US Food and Drug Administration) granted accelerated approval of trastuzumab deruxtecan for HER2-positive solid tumours [75].

TROP2 (trophoblast cell surface antigen 2) targeting ADCs have similarly shown benefit in a genomic-derived subgroup. TROP2 is a cell surface protein overexpressed in lung cancer, with low expression in normal lung tissue [76,77]. Second-line sacituzumab tirumotecan improved PFS and OS over pemetrexed plus carboplatin or cisplatin in EGFR-mutated non-squamous NSCLC in the recent OPTITROP lung-04 phase III trial. Overall, 376 patients underwent randomisation, median PFS was 8.3 months in the sac-TMT group and 4.3 months in the chemotherapy group (HR for disease progression or death, 0.49; 95% CI, 0.39 to 0.62), and 18-month overall survival was 65.8% and 48.0%, respectively [78]. However, other TROP2-directed ADCs have not demonstrated a clear survival benefit in the second-line setting, despite their promising phase I and II data. Datopotamab deruxtecan early-phase results in TROPION-PanTumor01 were encouraging, with an ORR of 26%, independent of TROP2 expression [79]. Despite this early promise, it did not translate into a significant OS benefit in later-phase trials compared with docetaxel. In the open-label, phase III TROPION-Lung01 trial, 299 and 305 patients were randomly assigned to receive datopotamab deruxtecan or docetaxel, respectively. In the intention-to-treat population, the median PFS was 4.4 months with datopotamab deruxtecan versus 3.7 months with docetaxel (HR, 0.75; 95% CI, 0.62–0.91; *p* = 0.004). Median OS was similar between the two groups, at 12.9 months and 11.8 months, respectively (HR, 0.94; 95% CI, 0.78–1.14) [80]. Adverse events of note include oral mucositis/stomatitis (55.2%), ocular surface events including lacrimation (7.7%), dry eyes (7.1%), keratitis (4.0%), and ILD (interstitial lung disease, 8.8%) [80]. In the multicenter, single-arm, phase II ICARUS-Lung01 study, datopotamab deruxtecan was administered to heavily pretreated patients with advanced NSCLC. The data suggest that patients with non-squamous NSCLC derive greater benefit than those with squamous NSCLC, with an ORR of 32.9% versus 5.0% and a median PFS of 4.8 versus 2.9 months, respectively [81]. Among the negative later-phase trials evaluating TROP2-targeting ADCs, EVOKE-01 compared sacituzumab govitecan with standard-of-care docetaxel in patients with previously treated NSCLC. The study did not meet its primary endpoint of OS, with a median OS of 11.1 months in the sacituzumab govitecan arm versus 9.8 months in the docetaxel arm (HR, 0.84; 95% CI, 0.68–1.04) [82].

One promising area of progress focuses on MET pathway dysregulation. c-Met is a cell surface receptor expressed in stem cells, the liver, and cancer stem cells, overexpressed in lung cancer, but not expressed in normal lung tissue [66,83,84]. Although *MET* exon 14 skipping mutations function as canonical drivers, many non–oncogene-addicted tumours display *MET* amplification or heightened protein expression [85,86,87], which can augment tumour growth and contribute to therapeutic resistance. ADCs targeting c-MET are being evaluated to exploit these expression patterns. In the single-arm, phase II LUMINOSITY study, the c-MET–directed ADC telisotuzumab vedotin showed activity in previously treated, *EGFR*-wildtype, non-squamous NSCLC with c-MET overexpression (defined as high if ≥50% of tumour cells with 3+ staining and intermediate if ≥25% and <50%). Median DOR was 8.3 months (9.0 months in the c-MET–high subgroup) and median OS was 14.5 months (14.6 months in the c-MET–high subgroup) [88]. An important toxicity was peripheral sensory neuropathy in 30% of patients [88]. Telisotuzumab vedotin is currently being evaluated in the ongoing TeliMET NSCLC-01 phase III trial, where previously treated c-Met-overexpressing locally advanced/metastatic non-squamous NSCLC patients are randomised 1:1 to telisotuzumab vedotin versus docetaxel [89].

Nectin-4 is another transmembrane receptor overexpressed in cancers, including NSCLC, yet minimally expressed in healthy tissue [90]. Preclinical work demonstrates that Nectin-4 stabilises and increases cell-surface CD155 (cluster of differentiation 155), promoting resistance to PD-1 blockade and enhancing TIGIT (T cell immunoreceptor with immunoglobulin and ITIM domains)-mediated immune suppression, whereas combined PD-1/TIGIT inhibition can overcome this effect [91]. Various ADCs targeting Nectin-4 in NSCLC are in development [90]. Additionally, zelenectide pevedotin, a Nectin-4–targeted bicyclic peptide conjugated to cytotoxic MMAE (monomethyl auristatin E), has produced encouraging activity in heavily pretreated NSCLC, particularly in patients with Nectin-4 amplification with increased response rates (ORR 40%), in early phase trials, with treatment-related adverse events (TRAEs) of clinical interest including peripheral neuropathy (33%), neutropenia (22%), and skin reactions (22%) [92,93].

Another cell surface biomarker of interest is IB6 (integrin beta 6), a member of the integrin family, and exists as a heterodimer with integrin alpha-v. Sigvotatug vedotin is an IB6-directed ADC in development, which has shown promise in a phase I study examining this drug in multiple advanced solid tumours, including NSCLC. In 113 NSCLC patients treated on this trial, the confirmed ORR was 19.5% (95% CI 12.6–28.0) in all patients with NSCLC, with particular benefit in patients with non-squamous, taxane-naïve NSCLC, where the reported ORR was 32.5% (95% CI, 18.6–49.1) [94]. The most common treatment-emergent adverse event of grade 3 or higher was dyspnoea in 9.7% of patients [94]. The phase III Be6A Lung-01 study is currently in progress, comparing sigvotatug vedotin versus docetaxel in patients with non-squamous NSCLC who have progressed on ≥1 line of platinum-based chemotherapy [95]. The ongoing phase III Be6A-Lung-02 study compares sigvotatug vedotin in combination with pembrolizumab versus pembrolizumab monotherapy as first-line treatment for patients with PD-L1 ≥ 50% advanced NSCLC [96]. Table 1 summarises the key antibody–drug conjugate trials.

### 3.2. Novel Immunotherapies

#### 3.2.1. Immune Checkpoint Inhibitors

Immunotherapy monotherapy or with PD-1/PD-L1 yields durable responses and long-term survival in up to 20% of NSCLC patients, sometimes extending OS beyond 3–5 years [25,97,98,99]. The subset of prolonged responders is, however, yet to be identified with biomarker selection beyond PD-L1, and acquired resistance usually ensues [100]. Resistance is typified by insufficient numbers and/or dysfunction of cytotoxic and/or memory T-cells [101,102,103,104,105]. These can be caused by insufficient neoantigen presentation [104], an immunosuppressive tumour microenvironment [106], ineffective intratumoural infiltration, impaired immune signalling pathways, metabolic dysfunction, alternative immune checkpoint ligands, T-cell exhaustion, or epigenetic alterations [101]. Attempts to overcome resistance now include next-generation checkpoint inhibitors (LAG-3 [lymphocyte-activating gene-3], TIM-3 [T-cell immunoglobulin and mucin domain-containing protein 3], TIGIT), bispecific antibodies, cytokine modulators, and vaccines [107,108,109]. Novel immunotherapy mechanisms emerging from early-phase programmes (i.e., TIGIT and LAG-3), as well as adenosine pathway blockade, show mixed signs of early promise, but targeting checkpoint inhibitors beyond PD-1 and CTLA-4 in NSCLC has demonstrated limited clinical benefit to date [107,110,111]. TIGIT suppresses T and NK (natural killer) cells and is overexpressed in NSCLC patients [112]. In the phase II CITYSCAPE trial, atezolizumab plus tiragolumab did not significantly improve median OS over placebo (23.2 versus 14.5 months, HR 0.69; 95% CI: 0.44 to 1.07) [107]. The larger phase III SKYSCRAPER-01 trial, tiragolumab plus atezolizumab versus placebo plus atezolizumab, likewise failed to meet its primary median OS endpoint (23.1 versus 16.9 months; HR 0.87; 95% CI: 0.71 to 1.08) [113]. Development of other TIGIT inhibitors, including vibostolimab and SGN-TGT, has been discontinued [114,115]. Although agents such as rilvegostomig [116], EOS-448, and domvanalimab remain under investigation, TIGIT targeting has not yet altered the standard of care for advanced NSCLC.

LAG-3 is an immune checkpoint expressed on exhausted T cells that inhibits T-cell proliferation and activation [117]. Although the LAG-3 checkpoint inhibitor relatlimab has demonstrated a survival benefit in randomised phase III trials in metastatic melanoma [118], its clinical activity in NSCLC remains unconfirmed. Currently available evidence is limited to exploratory post hoc subgroup analyses involving small patient numbers. To address this gap, the randomised phase III RELATIVITY-1093 trial is currently enrolling patients with metastatic non-squamous NSCLC and PD-L1 expression ≥ 1% to compare nivolumab plus relatlimab with platinum-doublet chemotherapy versus pembrolizumab plus chemotherapy [119]. Beyond relatlimab, several other LAG-3-targeted strategies are under investigation. In a phase II study of the anti-LAG-3 antibody ieramilimab combined with the anti-PD-1 agent spartalizumab, the ORR in advanced NSCLC was 15% in PD-1/PD-L1-naïve patients and 0% in previously treated patients [120]. Fianlimab combined with cemiplimab demonstrated encouraging activity, with an ORR of 50% in treatment-naïve patients and 26.7% in PD-1/PD-L1-naïve NSCLC, supporting further evaluation in an ongoing randomised phase II/III trial adding fianlimab to cemiplimab (anti-PD1) plus chemotherapy irrespective of PD-L1 status [121]. Among LAG-3-directed bispecific antibodies, the PD-L1/LAG-3 agent tobemstomig failed to improve PFS or ORR compared with pembrolizumab plus chemotherapy at interim analysis of a phase II trial [122], whereas tebotelimab (a dual targeting PD-1 and LAG-3 antibody) demonstrated an ORR of 14% (2/14; 95% CI: 2–43) in the immune checkpoint inhibitor-naïve NSCLC cohort and 0% (95% CI: 0–22) in the immune checkpoint inhibitor–refractory NSCLC cohort [123].

#### 3.2.2. Adenosine Targeting

The adenosine pathway is an emerging immunotherapy target in NSCLC due to its potent immunoregulatory effects within the tumour microenvironment. Adenosine is generated from extracellular ATP through the enzymatic activity of CD39 and CD73, leading to suppression of antitumor immune responses via adenosine receptor signalling [124]. Therapeutic strategies, therefore, focus on inhibiting CD73 or blocking adenosine receptors. Clinical proof of concept has been demonstrated with oleclumab, a CD73 inhibitor, in the phase II COAST trial, which enrolled patients with unresectable stage III NSCLC following chemoradiotherapy. The addition of oleclumab to durvalumab significantly improved PFS compared with durvalumab alone (not reached versus 6.3 months; HR 0.44; 95% CI: 0.26–0.75), without increasing severe toxicity (grade (G) ≥ 3 treatment-emergent adverse events 40.7% versus 39.4%) [111]. Complementary evidence comes from the ARC-7 phase II trial in treatment-naïve, PD-L1–high NSCLC, where triple blockade with zimberelimab (PD-1), domvanalimab (TIGIT), and the A2A receptor antagonist etrumadenant achieved an ORR of 40% and a median PFS of 10.9 months [125]. Other adenosine pathway modulators in development include uliledlimab (CD73 inhibitor) and ciforadenant and inupadenant (A2A receptor antagonists).

There is growing interest in developing T-cell engagers for NSCLC, driven in part by the clinical success of the bispecific T-cell engager tarlatamab in extensive-stage small cell lung cancer. A T-cell engager is an antibody that binds a T-cell and one (or more) of its targets, thus physically approaching them. This leads to T-cell activation, which can be further enhanced by additionally binding co-stimulatory molecules [126]. In NSCLC, multiple T-cell engager programmes are currently advancing through early phase I clinical trials, targeting a range of tumour-associated antigens, including mesothelin, EpCAM (epithelial cell adhesion molecule), and members of the claudin family [127,128]. These agents aim to redirect cytotoxic T cells toward malignant cells in a largely MHC-independent manner. However, systemic immune activation, such as cytokine release syndrome (CRS), and off-tumour toxicity remain key challenges. The main immunotherapy trials are summarised in Table 2.

#### 3.2.3. Bispecific Antibodies

Bispecific antibodies have two binding units, which can bind two antigens or epitopes at once [131]. Drug development in bispecific antibodies for non-oncogene-addicted NSCLC has progressed toward multi-pathway immunomodulation, particularly through dual-target constructs that combine immune checkpoint blockade with co-stimulation or angiogenesis inhibition and through conditional cytokine activation to amplify antitumour immunity while avoiding the systemic toxicities that historically limited cytokine-based therapeutics. Among these, PD-L1 × 4-1BB bispecifics such as acasunlimab (BNT311/GEN1046) have demonstrated clinically meaningful activity in early-phase studies. In the first-in-human phase I/II trial (NCT03917381) evaluating acasunlimab across advanced solid tumours, including PD-(L)1–refractory NSCLC, investigators reported a DCR of 65.6% (40/61 patients) with early radiographic responses and pharmacodynamic evidence of increased CD8^+^ effector memory T-cell expansion, IFN-γ induction, and enhanced NK-cell activation [132,133]. The most common TRAEs were asthenia (G ≥ 3 8.7%), diarrhoea (G ≥ 3 0%), nausea (G ≥ 3 0%), anaemia (G ≥ 3 4.3%), and liver-related events (G ≥ 3 8.7%). These results led to further study in NSCLC-specific cohorts: in a later phase II study (NCT05117242), acasunlimab combined with pembrolizumab yielded a 30% ORR (17% confirmed), 12-month OS of 69%, and median OS of 17.5 months, with adverse events largely low-grade, including manageable liver enzyme elevations (G ≥ 3 13%) [134]. Other toxicities were predominantly Grade 1–2 fatigue and infusion-related reactions, with limited high-grade immune-related events—demonstrating a favourable safety profile compared with historical 4-1BB agonists [135] and other cytokine modulators. In December 2025, Genmab announced the discontinuation of development of acasunlimab following a portfolio review to focus on higher-impact, late-stage opportunities [136]. Their phase II data suggest that targeting PD-L1 × 4-1BB remains a promising therapeutic strategy. In parallel, next-generation PD-1 × VEGF-A bispecifics such as ivonescimab have shown robust performance in non-oncogene-addicted disease; in the phase III HARMONi-2 trial for PD-L1-positive non-small cell lung cancer, median PFS was significantly longer with first-line ivonescimab than with pembrolizumab (11.1 vs. 5.8 months; HR 0.51; 95% CI: 0.38–0.69), including in the PD-L1 1–49% (HR 0.54; 95% CI: 0.37–0.78) and ≥50% (HR 0.48; 95% CI: 0.29–0.79) subgroups [137,138].

#### 3.2.4. Cancer Vaccines

Although earlier studies yielded modest results, accumulating data now indicate that cancer vaccines—especially those employing novel delivery platforms and rational patient selection—may meaningfully enhance immune responses in NSCLC. A recent meta-analysis of 11 phase II/III RCTs (n = 3228) showed that cancer vaccines administered after first-line therapy significantly improved overall survival (HR 0.85; 95% CI: 0.78–0.92) with acceptable safety, despite no clear PFS benefit, with particularly strong effects in patients with ECOG performance status 1, stable disease after first-line chemotherapy, smokers, stage IV disease, and notably squamous cell carcinoma [139]. Targeting molecular profiles further refines benefit; data from the phase I/II CIMAvax trial demonstrated superior outcomes with CIMAvax-EGF (recombinant human epidermal growth factor conjugated to a protein carrier) plus nivolumab in patients without driver mutations and with PD-L1 ≥ 1%, while Kirsten rat sarcoma viral oncogene (*KRAS)*-mutant tumours responded poorly [140]. CIMAvax-EGF has been well tolerated, as only 1% of 722 patients reported serious TRAEs. The most frequent adverse events regardless of causality were injection site pain (11.2%), dyspnoea (6.8%), fever (5.7%), chills (3.5%), headache (3.4%), and nausea (3.3%). Three patients (0.4%) had five serious related events consisting of anaphylactic shock, tremors (two events), redness of the upper limbs, vagal reaction, and chest pain [141]. Other molecular targets for NSCLC-directed vaccines currently in development include MAGE-A3, NY-ESO-1, and MUC1 [142]. Beyond tumour-specific vaccines, emerging evidence indicates that mRNA vaccines targeting non-tumour antigens, such as SARS-CoV-2, can act as potent immune modulators by inducing type I interferon responses, enhancing innate and CD8^+^ T-cell priming, and increasing tumour PD-L1 expression, thereby sensitising immunologically “cold” tumours to immune checkpoint inhibitors. Intriguingly, receiving a SARS-CoV-2 mRNA vaccine within 100 days of immune checkpoint inhibitor initiation has been associated with improved short- and long-term survival [143], even though immunotherapy does not seem to increase or decrease the immune response to the vaccine [144].

#### 3.2.5. Small Molecules

Small-molecule development for non-oncogene-addicted NSCLC has increasingly focused on targeting non-oncogene-addiction mechanisms, including AXL-driven immune suppression. AXL is a membrane receptor that, when activated by a ligand, activates the AXL signalling, which can lead to tumour immune tolerance and immune evasion [145]. Among the most clinically evaluated small molecules is bemcentinib, a highly selective oral AXL inhibitor, which was investigated as a strategy to counteract the stress-response and immune-evasion programmes commonly observed in biomarker-negative NSCLC. In the phase I bemcentinib + docetaxel trial (N = 21), previously treated NSCLC patients achieved 35% partial responses with a total disease-control rate exceeding 80%, despite high rates of grade ≥ 3 neutropenia (76%) [146]. Subsequent immunologic analyses showed that the combination of bemcentinib + pembrolizumab can actively reverse immune exhaustion hallmarks in chemo-immunotherapy-refractory lung adenocarcinoma, including reduction in M2-polarised macrophages, increased activation of type 1 conventional dendritic cells, and restoration of granzyme-B-expressing cytotoxic CD8^+^ T cells, with granzyme-B induction correlating positively with PFS [147]. This mechanistic evidence supports the concept that AXL blockade represents a non-oncogene addiction target, capable of restoring T-cell-mediated cytotoxicity and improving anti-PD-1 responsiveness even in PD-L1-negative or immune-excluded tumours. According to an analysis published in February 2025, BerGenBio officially terminated bemcentinib development, closing its final ongoing NSCLC study (BGBC016) after zero responses among 10 evaluable *STK11*-mutant patients, despite promising earlier phase I immunologic and clinical signals. This decision effectively ended the only late-stage small-molecule program targeting non-oncogene-addicted AXL-dependent NSCLC and underscores the translational difficulty of converting mechanistic non-oncogene targets into durable clinical benefit [148]. Despite discontinuation, the mechanistic and early clinical insights generated by bemcentinib trials—particularly its ability to reverse T-cell exhaustion and remodel suppressive myeloid compartments—remain foundational for future small-molecule strategies aimed at stress-response and immune-escape vulnerabilities in non-oncogene-addicted NSCLC. The main treatment-related adverse events with bemcentinib in combination with docetaxel were neutropenia (G ≥ 3 76%), diarrhoea (all grades 57%, G ≥ 3 0%), fatigue (G ≥ 3 5%), and nausea (all grades 52%, Grade ≥ 3 0%). Neutropenic fever occurred in 38% of patients.

### 3.3. Toxicities

Treatment-related toxicity is an important component in the decision to advance treatments in NSCLC to later-stage trials or into standard-of-care recommendations. Treatments may have various toxicities if used alone, and we can also see a synergistic effect when used in combination. The grade 3 or 4 toxicities of docetaxel chemotherapy are well documented. These most commonly include bone marrow suppression presenting as neutropenia (37.5%), thrombocytopenia (1.7%), anaemia (3.4%), gastrointestinal toxicity such as nausea (1.5%) and vomiting (1.0%), and peripheral sensory neuropathy (1.0%) [149]. With ADCs, side effects can be variable, relating to the mechanism of action of the payload. The most common side effects, as a class of drugs, are nausea (44.3%), neutropenia (43.8%), anaemia (37.7%), and alopecia (34.1%). Of significance, the overall incidence of ILD was 15.8%, and pneumonitis and ILD were the most frequent respiratory adverse events leading to discontinuation [150]. Immunotherapy toxicities include pneumonitis (G ≥ 3 1.8%), hypothyroidism (G ≥ 3 0.2%), nausea (G ≥ 3 0.8%), diarrhoea (G ≥ 3 0.6%), and infusion-related reaction (G ≥ 3 0.2%). These toxicities are less frequent than in chemotherapy but can be fatal. Immune checkpoint inhibitors, either as monotherapy or in combination, have been adopted in the standard of care for NSCLC. With this, we have seen the rise in immune checkpoint inhibitor-related pneumonitis, the most fatal toxicity of PD-(L)1 monotherapy [151]. Toxicities of novel agents are described in the preceding sections. As we move into an era of combination strategies of novel drugs in NSCLC, it is important to assess toxicity burden and quality of life versus the existing standard of care and the chronicity of toxicity that might not have been previously appreciated from clinical trial data.

## 4. Trial Design in Non-Small Cell Lung Cancer

Trial design has also seen advances in recent years, from phase I to phase III. In a phase I clinical trial, following preclinical testing, a molecule is dosed in participants. Here the optimal posology, i.e., the recommended phase II dose or dose range (RP2D), is established so its efficacy can later be assessed in phase II and III trials [152]. Phase I trials focus primarily on safety rather than efficacy, recognising that extensive pre-clinical studies may still incorrectly predict side effects and clinically effective doses [153,154].

Historically, when anti-cancer therapies were mostly chemotherapeutics, the RP2D would be based on the maximum tolerated dose (MTD) [155]. This was meant to maximise the new agent’s potential efficacy, given the direct dose–response relationship [156,157,158]. However, newer treatment modalities challenge this paradigm. Immunotherapy phase I trials usually cannot identify an MTD, because higher dosages do not correlate with further efficacy or toxicity (excluding the relatively dose-dependent anti-CTLA4 toxicity) [159]. Target therapy sometimes cannot find an MTD, and higher doses may start having off-target toxicities with no efficacy increase [156]. In these cases, the RP2D should consider other criteria. Phase I trials, therefore, include pharmacokinetic (PK) and pharmacodynamic (PD) evaluations, including serial blood draws following drug administration or pre- and post-treatment biopsies. Project Optimus, launched by the FDA in 2021, sets recommendations for dose-optimisation in clinical trials, considering the respective dose-effect and dose-toxicity curves of different treatment modalities [160,161]. It includes assessing various doses, trial designs with dose optimisation and dose confirmation, collaboration between industry, academia, professional societies, regulators, and patients, and model-based decision making [160,161,162,163]. This concern for drug activity also led to trial dose escalation design improvements. The classic “3 + 3” design can identify an MTD straightforwardly, but with inferior statistical performance and slow dose escalation, ignoring biological (in)activity. Newer designs can overcome these limitations, like Bayesian Optimal Interval (BOIN) or pharmacologically guided dose escalation (PGDE) [164,165].

Biological insights from cancer research have yielded more effective drugs, sometimes noticeable even in phase I trials. Pembrolizumab [166] and crizotinib [167] are examples where phase I trials achieved approval by the FDA due to pronounced benefits. Modern phase I trials are also searching for early signs of efficacy to accelerate approvals, using expansion cohorts and seamless phase I/II designs. Expansion cohorts often focus on specific histology or biomarkers to probe for efficacy signals [168,169,170], being associated with expedited and higher FDA approval rates [171,172]. Seamless phase I/II designs consolidate both phases into a single protocol, which can be quickly amended when needed and thus avoid the delays of approving a phase II protocol after phase I is complete [173]. These seamless designs can quickly open new cohorts for specific histological phenotypes or biomarker-positive tumours when promising data arise from the phase I part or other studies. These, nevertheless, have limitations, being non-randomised and frequently with unclear statistical analysis.

Phase III then ensues, directly comparing the new treatment against the current standard of care. If positive, it can change medical practice. However, using large phase III trials to test, one by one, new treatments for a cancer type or a single new treatment in different cancer types is a slow and costly process [174]. This motivated the creation of master protocols, which are trials efficiently testing multiple investigational agents simultaneously [175]. They improve efficiency by establishing common trial networks, infrastructure, data management, a central randomisation system, trial design, schedule of activities, procedures and assessments, outcomes, and a control group [175], with biomarker-defined sub-studies enabling more efficient “go/no-go” decisions [176]. Start-up after protocol expansions or new arm additions is also faster after repeat amendments rather than after design and approval of sequential individual clinical trial protocols [175]. Master protocols include umbrella, basket, and platform trials. An umbrella trial, like the Lung-MAP trial [177], tests different treatments (according to biomarkers) for a single disease type, often sharing a single control arm [174,175,178]. Basket trials are rare in phase III, as they test a single treatment in different populations [179]. Lastly, platform trials like CONCORDE [180,181] are usually phase III and test different treatments using a common control group, and can add new arms for promising new treatments or stop ineffective arms [182,183].

## 5. Why Do Promising Drugs in NSCLC Not Change Practice?

**Figure 3:**
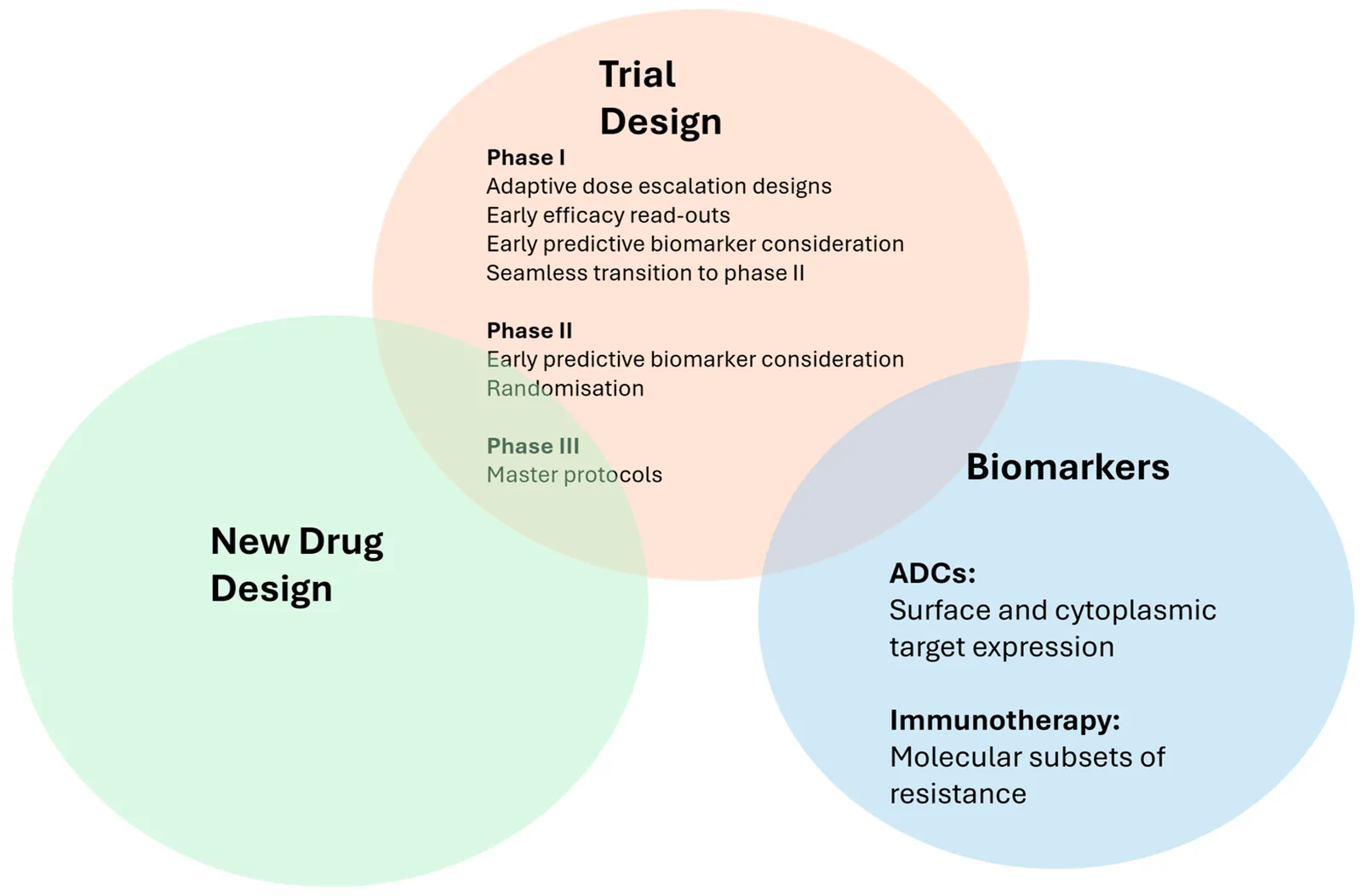
Drug developments in non-oncogene-addicted non-small cell lung cancer. Abbreviations: ADC: antibody–drug conjugates.

Unfortunately, most of the latest advances in NSCLC drug development ultimately fail to change current medical practice. While more than half of randomised phase III oncology trials fail to meet their primary endpoints [184], clinical trial success rates for advanced-stage NSCLC therapies are significantly lower than biopharmaceutical industry averages overall (11% versus 16.5%), mainly due to poor phase III performance [185]. Achieving a successful trial outcome in NSCLC poses several challenges; however, some potential solutions could be found in biomarkers (for ADCs, immunotherapies, or novel therapies), improved drug design, and better trial design, as discussed here (Figure 3).

### 5.1. Selection by Biomarkers

Biomarker-targeted NSCLC therapies show dramatically higher success rates across all phases, with a cumulative pass rate of 62% compared to 19% for industry aggregates [185]. Genomic biomarker-driven patient selection has reshaped drug development strategies for oncogene-addicted NSCLC, but selection by target in the case of non-oncogene-addicted NSCLC has not afforded a similar benefit to date.

#### 5.1.1. Antibody–Drug Conjugates

In the case of ADCs, expression of the target could reasonably be presumed to influence target engagement and thus efficacy. This, in practice, does not appear to be the case. As an example, in HER-2 mutant NSCLC (considered oncogene-addicted [186]), trastuzumab deruxtecan achieved remarkable response rates around 50% throughout the phase I to II trials that translated into regulatory approval [187,188,189]. These were in contrast with the ORR of around 30% when the same drug was used in non-oncogene-addicted NSCLC overexpressing HER2 in IHC [187,188,189,190]. TROP2 expression by IHC also did not correlate with response to sacituzumab-govitecan [191] or datopotamab deruxtecan [79], nor did genomic driver alterations with the latter [192]. Telisotuzumab vedotin response correlated qualitatively with c-MET positivity in IHC, but not in a quantitative linear way [193]. The same lack of correlation between IHC target expression and ADC response was described for integrin beta-6 and sigvotatug vedotin [194], HER3 and patritumab deruxtecan [195], and B7-H3 and HS-20093 [196]. Of note, CEACAM5 (carcinoembryonic antigen-related cell adhesion molecule) expression could be more of a prognostic biomarker rather than predictive for tusamitamab ravtansine [197]. It would thus seem that a high target expression might not be crucial for ADCs, perhaps because only a very small amount of target expression suffices. The lower limit of antigen expression required for effective targeting and anti-tumour activity is currently undefined and varies based on numerous factors related to the antibody, antigen, and tumour characteristics impacting sensitivity to the payload. Possible reasons for an ADC working in a context where monoclonal antibodies (mAbs) were unsuccessful are highly cytotoxic payloads, a high drug-to-antibody ratio, and potent bystander effects. Because of the bystander effect, after an ADC molecule is internalised and its payload released from the killed cell, it can have a cytotoxic effect on the adjacent tumour cells even if they do not express the target [198]. Immune responses can also be elicited by the ADC, especially if its antibody component, once separated from the payload, contains an Fc region that can activate immune cells [199]. Furthermore, the localisation of the target could be important, more so than its overall expression. The normalised membrane ratio (NMR) measures the target’s expression in the membrane relative to the whole cancer cell (membrane and cytoplasm), using IHC and Quantitative Continuous Scoring (QCS) [200]. TROP2 NMR positivity was predictive of higher ORR and longer PFS with datopotamab deruxtecan in an exploratory analysis of TROPION-Lung01 [200].

#### 5.1.2. Novel Immunotherapies

While a biomarker-based approach has not yet overcome immunotherapy resistance in immunotherapy-treated NSCLC, several intracellular and surface biomarkers, with profound effects on tumour biology and immune responsiveness, are emerging as therapeutic guideposts. Among the most prominent are *STK11*, *KEAP1*, and *SMARCA4* (SWI/SNF-related, matrix-associated, actin-dependent regulator of chromatin, subfamily A, member 4) loss—each defining molecular subsets with characteristic patterns of immune evasion or metabolic adaptation. *STK11* inactivation disrupts interferon signalling and limits T-cell infiltration [201], while *KEAP1* mutations activate NRF2 (nuclear factor erythroid 2-related factor 2)-driven antioxidant and metabolic programmes that create an immune-excluded microenvironment [202]. Loss of SMARCA4, a SWI/SNF (SWItch/Sucrose Non-Fermentable) chromatin-remodelling component, promotes dedifferentiation, absence of cell lineage fidelity, and impaired antigen presentation, with the most extreme phenotype manifesting as SMARCA4-deficient undifferentiated tumours [203]. Their clinical impact is further shaped by co-mutation contexts; for example, NSCLC with *KRAS*/*SMARCA4* class 1 co-mutations demonstrate strikingly poor OS compared to those with *KRAS* mutations alone (HR = 3.23, *p* < 0.001) [204]. Therapeutic strategies targeting these vulnerabilities—including CoREST (corepressor of RE1-silencing transcription factor) inhibition or STING (stimulator of interferon genes) activation for STK11 loss [205,206], glutamine metabolism inhibition for *KEAP1*-mutant tumours [207], and synthetic-lethal SMARCA2 inhibition for SMARCA4 deficiency [208]—are progressing through clinical development. A summary of emerging biomarkers in NSCLC can be found in Table 3.

### 5.2. Improved Drug Design

**Figure 4:**
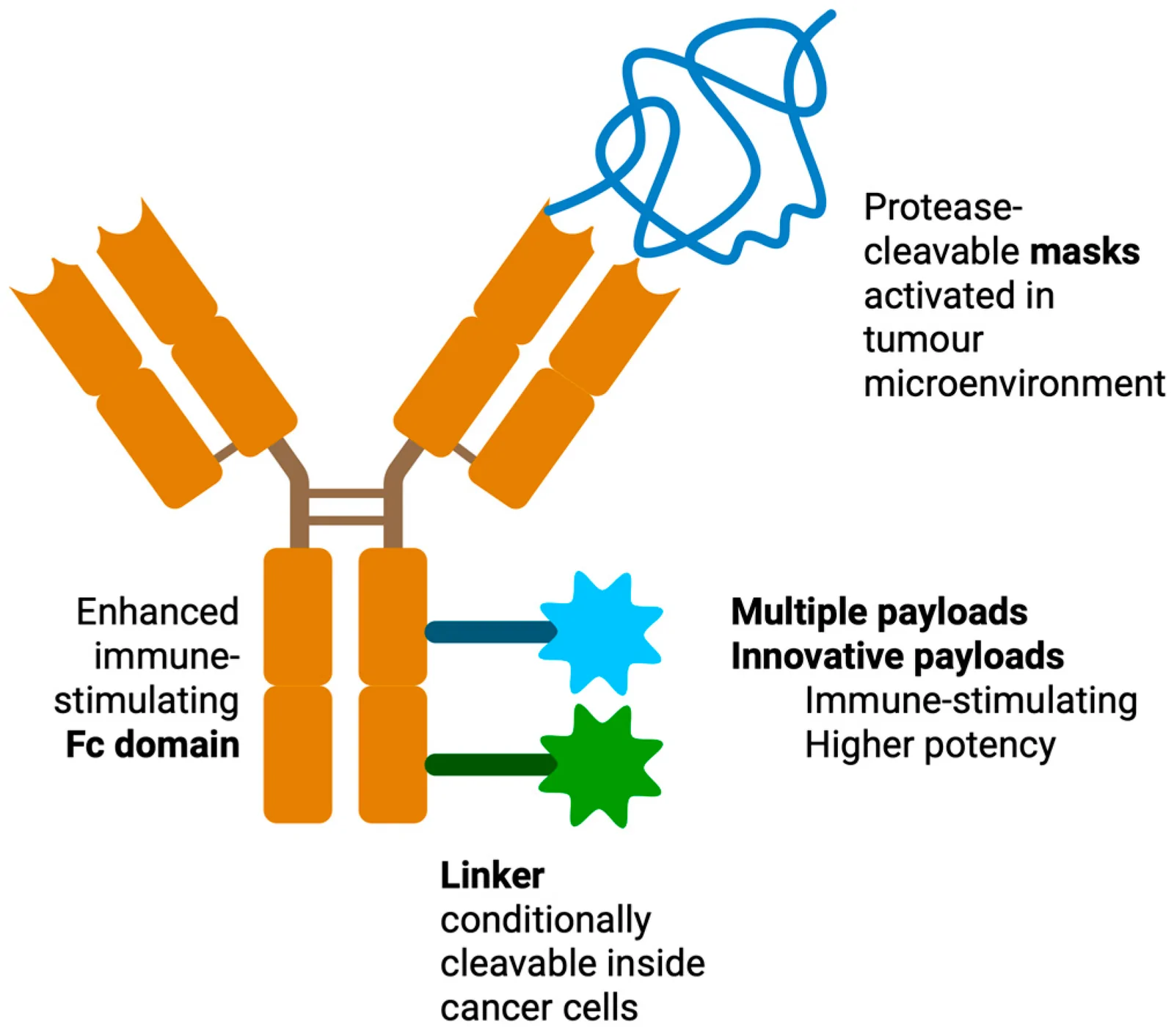
Developments in antibody–drug conjugate design. Created in BioRender. C, P. (2026) https://BioRender.com/9th3ygg (accessed on 3 March 2026).

Emerging technological innovations may improve the therapeutic success of ADCs (Figure 4). Next-generation ADCs incorporate dual-payload strategies aiming to deliver two synergistic cytotoxic agents, potentially overcoming resistance and enhancing efficacy [209,210], have refined linkers and payloads, and have innovative designs including conditional activity, bispecificity, multi-payloads, or enhanced immune stimulation [211]. Single- and double-masked T-cell engagers, incorporating protease-cleavable masks that are selectively activated within the tumour microenvironment, may mitigate on-target/off-tumour toxicities and improve PK, safety profiles, and the therapeutic window [113,126,212,213].

HER2 ADCs exemplify how drug design improved responses after an earlier iteration failed. Ado-trastuzumab emtansine (T-DM1), using a non-cleavable linker, which can reduce its bystander effect [56,214], showed only limited activity in NSCLC studies, with ORR ranging between 0% and 44%, median PFS between 2.6 and 6 months, and median OS between 2.0 and 15.3 months [56,214,215,216,217,218]. Later, [fam-] trastuzumab deruxtecan, using a cleavable linker and a new campthothecin payload with a higher drug to antibody ratio [214,219,220], achieved significant results, including ORR ranging from 42.9% to 72.7%, median PFS 8.2 to 11.3 months, and median OS 11.2 to 17.8 months, ultimately achieving FDA and EMA approval [187,188,190,221].

In the field of immune-oncology, there are many evolving targets, as discussed above. Enhanced drug design is also being applied to these antibody-based therapies, including T-cell engagers. These include reducing CRS by intra-patient step-up dosing or by lowering CD3 affinity, extending drug half-life by adding albumin as a specific target in tri-specific antibodies, or by incorporating an Fc domain, and minimising on-target side effects through protease- or pH-activated binding domains [126].

### 5.3. Improved Trial Design

The drug development process in NSCLC, as in other solid tumours, needs to be robust, seeking early signals of efficacy and, equally, defining futility so that drug development efforts can be channelled early into those drugs more likely to ultimately improve patient outcomes.

Phase I clinical trials’ evolution from toxicity-defining studies to “seamless” phase I/II designs with therapeutic intent has reshaped health policy considerations for NSCLC [222].

In this setting, disease heterogeneity and predominant reliance on immune checkpoint inhibitors mean that early efficacy signals, often based on ORR in small, highly selected cohorts, frequently correlate poorly with OS [186,223]. As a result, regulatory agencies must balance the pressure of fast-tracking drug approval for an unmet need population against the risk of approving new drugs based on unvalidated surrogate endpoints in a population where chemo-immunotherapy is already an established, effective standard of care [223,224]. Recognising these limitations, contemporary regulatory frameworks are progressively shifting toward incorporating randomisation early in the drug development process for non-oncogene-addicted NSCLC [222,224]. While single-arm data may remain acceptable for breakthrough or accelerated approval in rare, high-response, molecularly defined subsets, they are increasingly viewed as insufficient in a broadly defined, non-oncogene-addicted NSCLC biologically heterogeneous population [223,224]. This is supported by experience with expansion cohorts in phase I trials, which increase the probability that a new drug will proceed successfully into phase II and ultimately obtain approval, but also highlights that high response rates alone do not guarantee phase III survival benefits [222,224]. Therefore, regulatory agencies have begun to privilege master protocol strategies as the preferred bridge between early and confirmatory phases in non-oncogene-addicted NSCLC [176,225]. In this context, mandatory biomarker stratification and prospectively planned randomised control arms introduced earlier in development are increasingly regarded as necessary to separate true treatment effect from prognostic or selection bias [176,186,225]. Adaptive designs, which allow pre-specified modifications based on interim analyses (such as “go”/“no go” decisions based on predefined efficacy bars), can minimise exposure to ineffective therapies while accelerating the progression of promising agents [226]. Other trial designs are also emerging, including for addressing cancer evolution [227]. Incorporating robust translational endpoints—such as dynamic biomarker validation, pharmacodynamic readouts, and early immune signatures—may enable more accurate prediction of long-term benefit beyond ORR [228,229]. Regulatory harmonisation efforts converge on the principle that, in non-oncogene-addicted NSCLC, early therapeutic benefit in phase I trials must be validated through robust comparative evidence and biomarker quality assurance before broad approval [176,223,225]. This translates into higher evidentiary standards for accelerated pathways, stronger requirements that confirmatory trials be underway at the time of conditional approval, and better validation of surrogate endpoints by disease context [224,230]. Ultimately, these policy shifts aim to reduce late-stage attrition and ensure that apparent benefit in phase I translates into reproducible, population-level survival gains while preserving timely access for patients with highly unmet needs [224,230].

## 6. Placing Drugs—First- or Second-Line?

Another possible way of improving the NSCLC trial success rate is to reconsider the positioning of new drug trials in first- or second-line settings, as the modest efficacy seen with second-line docetaxel appears to be a difficult hurdle for newer agents to clear. Docetaxel is the standard second-line option for NSCLC based on a randomised prospective trial comparing docetaxel to best supportive care, with an ORR of 13.1% [231]. This trial was done prior to immunotherapy being given in a first-line setting, but docetaxel remains the recommended second-line treatment option. Notable examples of negative phase III trials against docetaxel have already been discussed.

If new drugs do not improve outcomes in the second-line setting, this leads us to question whether they would be better placed in the first-line setting, in combination, or in place of standard first-line therapies. Causes of failure in late-phase trials can include unmodifiable factors such as inadequate understanding of therapeutic pathways [232] or heterogeneity in tumour biology [229]. In this setting, moving the late-phase trial to a first-line setting may not necessarily improve outcomes, as while the resultant ORR and PFS may be higher in a first-line population in comparison to a later-line population, this may not be improved relative to the current first-line standard of care. Also, “failures” against docetaxel should be keenly appraised, as modifiable factors can include suboptimal study design choices in patient population, biomarker selection, and analysis of outlier responders to improve on patient selection in future efforts [232].

There may, however, be good biological reasons for placing drugs earlier in the patient journey, as the immune environment could be different in a first- and second-line setting. Most of the immunotherapy resistance mechanisms discussed above can become more expressive later during the course of the disease. Such could contribute to a more challenging immune environment when testing drugs in a second-line setting instead of a first-line. Mutations and epigenetic changes accumulate in tumour cells, impairing neoantigen formation, processing, and presentation [106,233,234], interferon effector pathways [233,234], and ultimately the immune response [227,235,236,237,238,239]. Long-standing inflammation can cause higher concentrations of immunosuppressive cytokines or metabolites [101,104,240,241]. The alternative co-inhibitory immune checkpoint TIM-3 may be associated with adaptive resistance to PD-1 blockade [242,243]. Furthermore, CD8-positive T cells can become dysfunctional over time due to chronic exposure to tumour antigens, further hampering the immune system in late lines [244]. It is conceivable that mixed responses or oligometastatic progressions could result from the immune environment starting to change in some of the lesions [101]. In consideration of this, a possible strategy in selecting drugs that would benefit from a first-line setting would involve an assessment of how their mechanism of action is impacted by changes in the immune environment. Novel immunotherapies and drugs designed to reverse or prevent immunoresistance may benefit from testing in an earlier line prior to modifications to the immune environment or after early progression on immunotherapy.

Therapy sequencing is also important for ADCs, as it can impact overall PFS and OS, as trials in other tumour types have shown [245]. In particular for ADCs, recent retrospective cohort studies in breast cancer have suggested that median time to failure is longer for the ADC used first than for the ADC used afterward, for either sacituzumab govitecan following trastuzumab deruxtecan or trastuzumab deruxtecan following sacituzumab govitecan [246]. This raises questions regarding the importance of the sequence order of ADCs to achieve the best OS, which in turn brings several challenges to first-line studies. A positive study would raise questions as to the ideal sequencing of therapy with the current first-line standard of care. Conversely, a negative study would also raise questions regarding future development of the new drug and if further trials should then be attempted in a later-line setting.

There are some practical concerns around standard-of-care comparators when considering first-line trials in NSCLC. The current first-line standard of care for non-oncogenic addicted NSCLC involves a platinum doublet with anti-PD-(L)1 therapy. Though pembrolizumab alone is licensed for those with adenocarcinomas and PD-L1 expression > 50%, emerging data suggest chemotherapy–immunotherapy combination may offer longer survival and broader benefit even in PD-L1 high patients [247], with potential exception for very high expressors (PD-L1 ≥ 90%) [248]. Thus, the choice of a comparator arm in PD-L1 high cohorts needs careful consideration of whether it should be pembrolizumab alone or in combination with chemotherapy. For patients with non-squamous metastatic NSCLC, consideration would also need to be given to the use of maintenance pemetrexed, which has established PFS and OS benefit [249].

Equipoise remains an important ethical consideration in the conduct of trials: balancing the drive for drug development with the participants’ health. The current first-line standard of care for NSCLC provides a good response rate with the possibility of a durable response. By contrast, in the absence of strong signalling from the early phase setting, it is arguable that equipoise could not be achieved with a new drug in comparison to the standard of care. Notably, even if it is within expert opinion that equipoise is present, differences in opinion among the general clinician and patient population may result in a potential barrier to trial participation [250].

## 7. Conclusions

Important advances in drug development for non-oncogene-addicted NSCLC have been seen in recent years. Innovative antibody–drug conjugates and immunotherapies are showing encouraging results, with some of them currently undergoing phase III trials. Trial design has also seen improvements that maximise an experimental drug’s chances to reveal its potential.

Yet, challenges remain, and ultimately, second-line options after immunotherapy remain limited as new drugs face high attrition when moving to late-phase trials in second-line against docetaxel. Consideration of the dynamic evolution of cancer in the context of exposure to prior agents, including immunotherapy, is required when considering where to position novel agents and combination strategies that consider resistance likely to be required for effective outcomes. The more long-term toxicity burden on patients with novel combinations may become a prominent consideration when positioning drugs early in a patient’s treatment journey. Predictive biomarker identification has been challenging in this patient group but should not be abandoned in future drug development efforts, having the potential to improve outcomes and spare toxicity to patients in cases of futile treatment.

The improvements seen with oncogene-addicted NSCLC show there can be a great shift in outcomes when we have greater opportunities to personalise treatment recommendations. There are constant improvements in biomarkers, drug design, and trial design, which are showing interesting results, and NSCLC drug development is looking promising.

## Figures and Tables

**Table 1:** Key antibody–drug conjugate trials in NSCLC.

Drug	Trial	Population	Overall Response Rate	Progression Free Survival	Overall Survival	Main Treatment Related Adverse Events Grade ≥ 3	Reference
Trastuzumab deruxtecan	DESTINY-Lung02	Previously treated metastatic HER2-mutant NSCLC	49%	9.9 months	19.5 months	Interstitial lung disease in 2.8%	[68]
Trastuzumab deruxtecan	DESTINY-PanTumor02	Previously treated advanced HER2 IHC 3+ tumours	61.3%	11.9 months	21.1 months	Neutropenia in 10.9% Anaemia in 10.9% Pneumonitis in 1.5%	[74]
Sacituzumab tirumotecan	OPTITROP lung-04	Previously treated EGFR-mutated non-squamous NSCLC	60.6%	8.3 versus 4.3 months; HR 0.49; 95% CI: 0.39 to 0.62	Not reached versus 17.4 months; HR 0.60; 95% CI: 0.44 to 0.82	Neutropenia 39.9%	[78]
Datopotamab deruxtecan	TROPION-PanTumor01	Previously treated advanced or metastatic NSCLC (with or without actionable genomic alterations)	26.4% versus 12.8%	4.4 versus 3.7 months; HR 0.75; 95% CI: 0.62 to 0.91	12.9 versus 11.8 months; HR 0.94; 95% CI 0.78 to 1.14]	Stomatitis in 6.7% Anaemia in 4.0% Pneumonitis in 3.7%	[80]
Datopotamab deruxtecan	ICARUS-Lung01	Previously treated advanced NSCLC	28.0%	3.6 months	11.9 months	Stomatitis in 10.0%	[81]
Sacituzumab govitecan	EVOKE-01	Metastatic NSCLC with progression on/after platinum-based chemotherapy, anti-PD-(L)1, and targeted therapy for actionable alterations	13.7% versus 18.1%	4.1 versus 3.9 months; HR 0.92; 95% CI: 0.77 to 1.11]	11.1 versus 9.8 months; HR 0.84; 95% CI: 0.68 to 1.04	Neutropenia in 24.7% Fatigue in 12.5% Diarrhoea in 10.5%	[82]
Telisotuzumab vedotin	LUMINOSITY	Previously treated, high MET expression, EGFR-wildtype non-squamous NSCLC	28.6%	5.7 months	14.5 months	Peripheral sensory neuropathy in 7.0% Increased alanine aminotransferase in 3.5% Pneumonitis in 2.9%	[88]
Zelenectide pevedotin	Duravelo-1	Advanced/metastatic solid tumours associated with Nectin-4 expression	24%	3.6 months	Not reported	Peripheral sensory neuropathy in 2% Neutropenia in 16% Fatigue in 6%	[92]
Sigvotatug vedotin	SGNB6A-001 trial	Advanced NSCLC	19.5%	3.5 months	Not reported	Dyspnoea in 9.7% Fatigue in 7.1% Neutropenia in 5.3%	[94]

Abbreviations: CI: confidence interval; EGFR: epidermal growth factor receptor, G: grade, HER2: human epidermal growth factor receptor, HR: hazard ratio, IHC: immunohistochemistry, NSCLC: non-small cell lung cancer.

**Table 2:** Key immunotherapy trials in NSCLC.

Drug(s)	Trial Name/Identifier	Population (NSCLC Setting)	Overall Response Rate	Median Progression Free Survival	Overall Survival	Main Treatment Related Adverse Events Grade ≥ 3	Reference
Atezolizumab + tiragolumab	CITYSCAPE	Chemotherapy-naïve, PD-L1 ≥ 1%, recurrent or metastatic NSCLC with no EGFR or ALK alterations	31.3% versus 16.2%	5.4 versus 3.6 months; HR 0.57; 95% CI: 0.37 to 0.90	23.2 versus 14.5 months; HR 0.69; 95% CI: 0.44 to 0.07	Lipase increase in 9%	[107]
Atezolizumab + tiragolumab	SKYSCRAPER-01	First-line advanced PD-L1-high, EGFR/ALK wildtype NSCLC	45.8% versus 35.1%	7.0 versus 5.6 months; HR 0.78; 95% CI: 0.63 to 0.97	23.1 versus 16.9 months; HR 0.87; 95% CI: 0.71 to 1.08	Immune-mediated adverse events in 16.1%	[113]
Ieramilimab + spartalizumab	NCT02460224	Advanced NSCLC PD-(L)1 inhibitor naïve	15%	3.9 months	Not reported	Arthralgia (any grade) in 23.9%	[129]
Fianlimab + cemiplimab	NCT05800015	Unresectable stage IIIB/IIIC or stage IV, PD-(L)1 naïve NSCLC	50%	2.6 months	Not reported	Treatment-emergent adverse events in 33.3%	[130]
Tobemstomig and chemotherapy	NCT05775289	Previously untreated, locally advanced, unresectable or metastatic NSCLC	41.1% versus 44.0%	7.6 versus 7.1 months; HR 0.99; 95% CI: 0.63 to 1.56	Not reported	Immune-related adverse events (any grade) in 67.4%	[122]
Tebotelimab	NCT03219268	Previously treated, checkpoint inhibitor NSCLC	14%	Not reported	Not reported	Rash in 1.5%	[123]
Zimberelimab + domvanalimab + etrumadenant	ARC-7	Treatment-naïve, PD-L1-high metastatic NSCLC	40%	10.9 months	Not reported	Treatment-emergent adverse events in 47%	[10]

Abbreviations: ALK: anaplastic lymphoma kinase; EGFR: epidermal growth factor receptor; CI: confidence interval; HR: hazard ratio; NSCLC: non-small cell lung cancer; PD-L1: programmed death-ligand 1.

**Table 3:** Emerging biomarkers in NSCLC: prevalence, biological mechanisms, and therapeutic implications.

Biomarker/Alteration	Prevalence in NSCLC	Biological Effect/Mechanism	Therapeutic Implications
STK11 (loss of function)	15–20%	Suppresses interferon signalling, reduces T-cell infiltration, creates immune-inert microenvironment	CoREST inhibition; STING activation; exploration of CTLA-4 + PD-(L)1 combinations
KEAP1 (loss of function)	15–20%	Activates NRF2 pathway, leading to metabolic rewiring, antioxidant-rich, immune-excluded niche	Glutamine metabolism inhibitors; combinatorial immunotherapy approaches
SMARCA4 (loss of function/deficiency)	5–10%	Chromatin-remodelling defect, leading to dedifferentiation, low antigen presentation; severe cases, leading to SMARCA4-UT	Synthetic-lethal SMARCA2 inhibitors (LY4050784, PRT3789)
KRAS + SMARCA4 co-mutation	—	Synergistic aggressiveness and lineage plasticity	KRAS-targeted therapies + biomarker-driven co-targeting strategies
HER2 overexpression	13–20%	Receptor overactivation	HER2 ADCs (trastuzumab deruxtecan)
TROP2 expression	~70–90%	Cell-surface target with internalisation properties	TROP2 ADCs (sacituzumab govitecan; datopotamab deruxtecan)
MET overexpression	~30% (high ~15%)	c-MET activation, leading to increased proliferation and survival	MET-directed ADCs (telisotuzumab vedotin)
Nectin-4 overexpression/amplification	>60% overexpressed; ~20% amplified	Stabilises and increases cell-surface CD155, promoting TIGIT-dependent immunosuppression and resistance to PD-1 blockade; functions as a high-internalisation drug–conjugate target	Nectin-4-directed ADCs/BTCs, TIGIT + PD-1 combination strategies

Abbreviations: ADC: antibody–drug conjugates, BTC: bicycle toxin conjugate, CD155: cluster of differentiation 155, CoREST: corepressor of RE1-silencing transcription factor, CTLA-4: cytotoxic T-lymphocyte associated protein 4, HER2: human epidermal growth factor receptor 2, KEAP1: Kelch-like ECH-associated protein 1, KRAS: Kirsten rat sarcoma viral oncogene, NRF2: nuclear factor erythroid 2-related factor 2, NSCLC: non-small cell lung cancer, PD-1: programmed cell death-protein 1, PD-(L)1: programmed cell death(-ligand) 1, SMARCA4: SWI/SNF-related, matrix-associated, actin-dependent regulator of chromatin, subfamily A, member 4, STING: stimulator of interferon genes, STK11: serine/threonine kinase 11, TIGIT: T cell immunoreceptor with immunoglobulin and ITIM domains, TROP2: trophoblast cell surface antigen 2.

## Data Availability

No new data were created in this study. Data sharing is not applicable to this article.
