# Correction: Cruz et al. Drug Development in Non-Oncogene-Addicted Non-Small Cell Lung Cancer. *Cancers* 2026, *18*, 880

**DOI:** 10.3390/cancers18142361

**Published:** 2026-07-22

**Authors:** Pedro Cruz, Cristina Boixareu, Diogo J. Silva, Joshua Ting, Rayssa Sena, Steph A. Pang, Stephanie Mullings, Anna Minchom

**Affiliations:** Drug Development Unit, The Royal Marsden Hospital and The Institute of Cancer Research, London SM2 5GP, UK

## Text Correction

During publication, the authors detected the following errors in this article [1]: reference 189 and references 191–200 in the corrected publication were mistakenly not included in the original publication, instead being misplaced by other references. With this correction, the order of references has been adjusted accordingly. The authors state that the scientific conclusions are unaffected. This correction was approved by the Academic Editor. The original publication has also been updated.
